# This one ritual

**DOI:** 10.1017/S1478951525100539

**Published:** 2025-09-12

**Authors:** Tara P Menon

**Affiliations:** Virginia Tech Carilion School of Medicine, Roanoke, VA, USA

her lips split at the edges
like overripe fruit
the corners crusted/not just dry,
but caked with old lemon glycerin
and days without appetite
someone says “just a quick swab,”
but i’ve learned this is the one ritual
to not rush

the pink sponge squeaks
against her back molars
she winces
not from pain
but from memory
grapejuice, communion wine,
a mouthful of coins for the river

morphine lingers like a film not a flavor
i clean her teeth like a daughter would
not sterile, not gentle
but right
the way you wipe lipstick
from someone you love
after they’ve fallen asleep in it

she hasn’t spoken in days
but when i finish,
she swallows

